# Integrin-linked kinase regulates cadherin switch in bladder cancer

**DOI:** 10.1007/s13277-016-5354-x

**Published:** 2016-09-28

**Authors:** Dorota Gil, Dorota Ciołczyk-Wierzbicka, Joanna Dulińska-Litewka, Piotr Laidler

**Affiliations:** Chair of Medical Biochemistry, Jagiellonian University Medical College, ul.Kopernika 7, 31-034 Kraków, Poland

**Keywords:** Cadherin, ILK, Snail, Twist, Zeb, EMT

## Abstract

Cadherin switch is specific of epithelial-mesenchymal transition (EMT) and is closely related to tumor cell invasion. However, the molecular mechanism that promotes the phenotypic changes remains unclear and elusive. We found that integrin-linked kinase (ILK) is a key factor involved in cadherin switch. The expression and activity of ILK are elevated in a variety of cancers but its mechanisms are not exactly understood. In this report, we studied the role and mechanism of ILK in EMT of human bladder cancer. We showed that silencing of ILK expression by small interfering RNA (siRNA) significantly abolished the nuclear translocation or the presence of markers associated with EMT like Snail, Twist, Zeb, and beta-catenin. ILK knockdown by siRNA suppressed N-cadherin expression and increased re-expression of E-cadherin in bladder cancer cells. We suggest that ILK is a major signaling factor involved in EMT. It is essential to understand the molecular mechanism of EMT in aim to possibly use it in search for new therapeutic targets.

**Electronic supplementary material:**

The online version of this article (doi:10.1007/s13277-016-5354-x) contains supplementary material, which is available to authorized users.

## Introduction

Bladder cancer is the ninth most frequently diagnosed cancer and the 12th leading cause of deaths worldwide. It is the second most common urinary tract malignancy and the fifth most common cancer in men and the 19th in women [1]. Of these tumors, 95 % are transitional cell carcinoma (TCC). Muscle invasive bladder cancer is related with high frequency of metastasis. Approximately 80 % of bladder cancers are nonmuscle invasive bladder cancer that rarely progress, and patients have good prognosis, but 30 % of those tumors progress into more aggressive and lethal forms. It is important to understand the molecular mechanism of bladder cancer metastasis to prevent cancer’s spread or to detect new therapeutic targets.

Epihtelial-mesenchymal transition (EMT) is a process by which epithelial cells lose their epithelial properties and obtain a mesenchymal phenotype. Tumor cells undergo epithelial to mesenchymal transition which transforms them from a quiescent cancer cells to a malignant phenotype. Loss of E-cadherin expression and induction of N-cadherin expression are a hallmark of the EMT process, which is needed for epithelial cells to adopt mesenchymal characteristic, a process also known as the cadherin switch [2]. A reduction or loss in expression of E-cadherin has been associated with bladder tumorigenesis. Supression of E-cadherin expression by transcriptional factors, including Snail, Twist, and Zeb, is engaged in various malignancies.

We have previously demonstrated that EMT markers in melanoma cells are dependent on ILK function [3]. ILK is a multifunctional intracellular effector of cell-matrix interactions and controls many cellular processes, including proliferation, survival, differentiation, migration, and invasion. ILK is a serine-threonine protein kinase which interacts directly with cytoplasmic domains of the β_1_ or β_3_ integrin subunits [4]. ILK coordinates several signaling pathways. In particular, it phosphorylates and activates Akt at Ser 473 which controls the genes essential for survival [4]. ILK can also directly phosphorylates GSK-3β at Ser 9, inactivate it, and lead to activation of some transcription factors [5]. Overexpression of ILK leads to downregulation of E-cadherin and nuclear accumulation of β-catenin and NF-κB activating the expression of other mesenchymal genes [3, 5–7]. The mechanism by which ILK induces the loss of E-cadherin and the progression of EMT is still unexplored, but current data suggest that ILK transcriptionally regulates Snail [8] or through Poly (ADP-ribose) polymerase-1 (PARP-1), [9] an unknown mechanism.

The mechanism associated with the role of ILK in tumor progression of bladder cancer is not well understood. Gao et al. [10] presented that ILK is involved in bladder cancer cell proliferation, growth, and apoptosis, and Matsui et al. [11] suggest that ILK expression is up regulated in invasive bladder cancer and plays a significant role in the EMT of bladder cancer by the control of E-cadherin and MMP-9 expression. They indicate that ILK regulates the EMT of bladder cancer and the mechanism depends on cell types. Although ILK is believed to be a key factor in EMT, its role in bladder cancer progression is not completely understood. The aim of the present study was to elucidate the mechanism of ILK-induced EMT and cadherin switch as a hallmark, in bladder cancer cells.

## Materials and methods

### Cell culture

The studies were carried out on two human cell lines: HCV29 (nonmalignant transitional epithelial cells of the urether, ATCC) and T24 (transitional cancer cells of the urine bladder, ATCC). Cells were cultured in RPMI-1640 medium supplemented with 10 % fetal bovine serum and penicillin/streptomycin in 95 % air and 5 % CO_2_ atmosphere.

### ILK knockdown using siRNA

Bladder cells were grown until 60 % confluence was reached and then transfected using INTERFERin™ according to the manufacturer’s protocol (Polyplus Transfection) with three different 21 bp double-stranded small interfering RNA (siRNA) molecules specifically targeting the ILK (Ambion ID#288570; ID#145116; ID#145117) or a control nonsilencing sequence (Ambion). Both cell lines were in each case transfected with 60 nM siRNA.

### Preparation of cytoplasmic, membrane, nuclear, and cytoskeletal cell lysates

Cytoplasmic, membrane, nuclear, and cytoskeletal extracts were prepared using the ProteoExtract® Subcellular Proteome Extraction Kit (MERC Millipore) according to producer’s protocol, and afterwards, equal amounts of protein were used for immunoblot analysis.

### Western blot analysis

Cell lysis and western blot were carried out as we previously described [3]. Antibodies for ILK, Akt, E-cadherin, Vimentin, GSK-3β, phospho-GSK-3β (Y216), β-catenin (all Transduction Laboratories, BD), phospho-Akt (S473), phospho-GSK-3β (S9), phospho-β-catenin (S552), phospho-β-catenin (T41, S33,37) (all Cell Signaling Technology), N-cadherin (R&D) and β-actin, ZEB1, TWIST1 (all Sigma) SNAIL (ABGENT), PARP-1, Calnexin, and HSP-90 (all Calbiochem) were used to detect indicated proteins.

### RNA extraction, cDNA synthesis, and RT-PCR analysis

Total RNA was isolated using RNeasy Kit (Qiagen) as per the manufacturer’s protocol. One microgram of total RNA was used for reverse transcription using Omniscript (Qiagen). The RT-PCR amplification was performed accordingly to previous method [3].

## Results

### Knockdown of ILK decreases Akt kinase activity

**Fig. 1:**
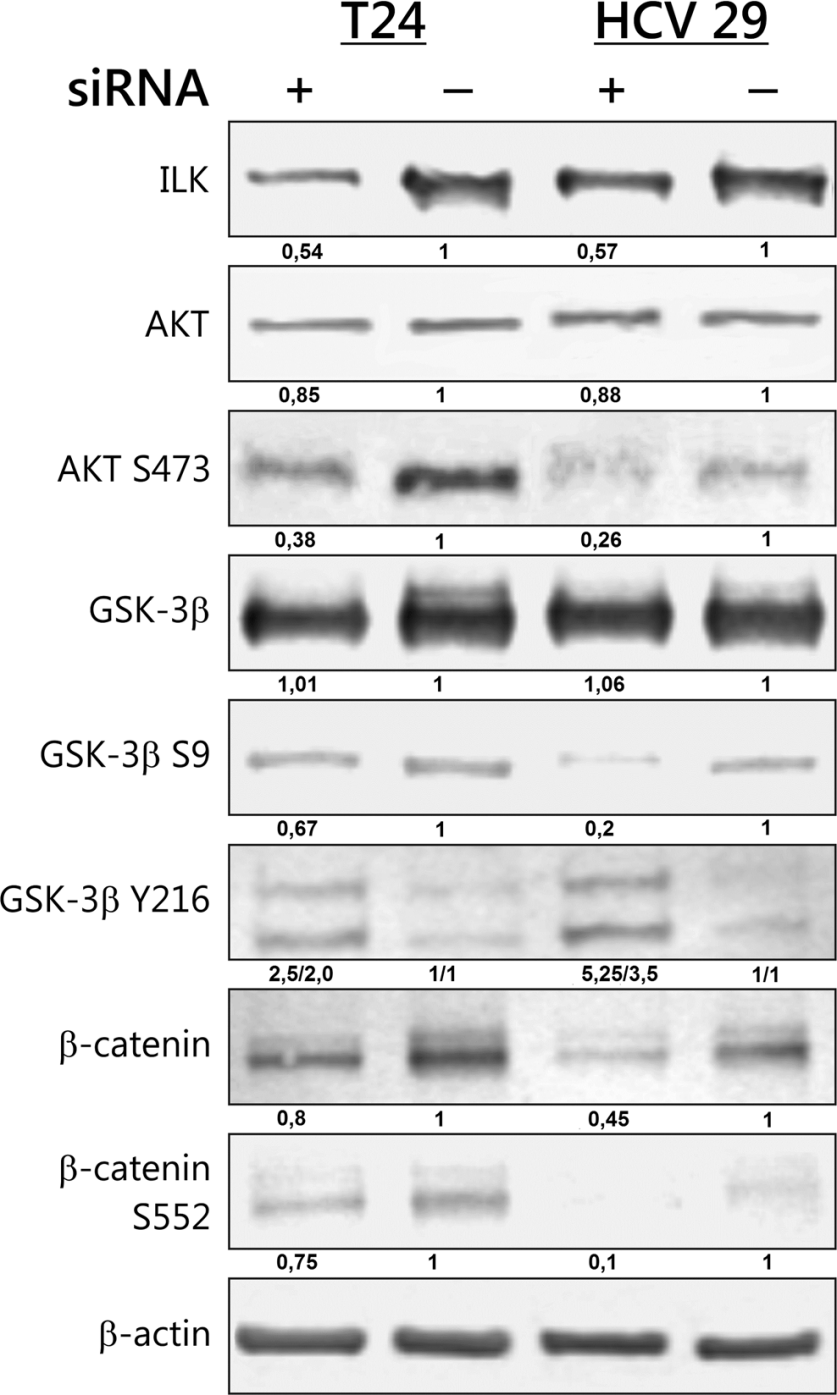
Knockdown of ILK inhibited activity of downstream signaling targets protein: Akt, GSK-3β, and β-catenin. Bladder cells were transfected with nonsilencing control siRNA or three different 21 bp double-stranded siRNA targeting the ILK. Forty-eight hours after transfection, protein expression was analyzed by Western blot. Total protein loading was determined by probing the membranes for β-actin. Densitometry was used to normalize to β-actin protein level and for quantitative comparison after siRNA knockdown. Presented are representative membranes of at least three independent experiments with similar results

Akt kinase is activated by phosphorylation at two sites, T308 in the kinase domain and S473 in the regulatory tail, and Akt is one of ILK targets. Expression of activated Akt was monitored by phospho-specific antibodies against phospho-Akt (S473). The results indicated that ILK in both bladder cell lines could activate Akt throughout the increase of its phosphorylation as knockdown of ILK expression using ILK siRNA, resulted in unanimous reduction of Akt activation with slight difference in its total expression (Fig. 1).

### ILK knockdown activates GSK-3β

GSK-3β activity is inhibited by direct phosphorylation at Ser 9. On the other hand, GSK-3β also possesses a tyrosine phosphorylation site (Tyr 216) that upon phoshporylation increases its activity [12]. siRNA silencing of ILK was sufficient for the activation of GSK-3β because the phosphorylation of GSK-3β on Tyr 216 was significantly higher, while phosphorylation on Ser 9 was markedly lower compared with control nonsilencing RNA-treated bladder cells (Fig. 1). There was however no change in the expression of total GSK-3β.

### ILK regulates the transcriptional activity of β-catenin

**Fig. 2:**
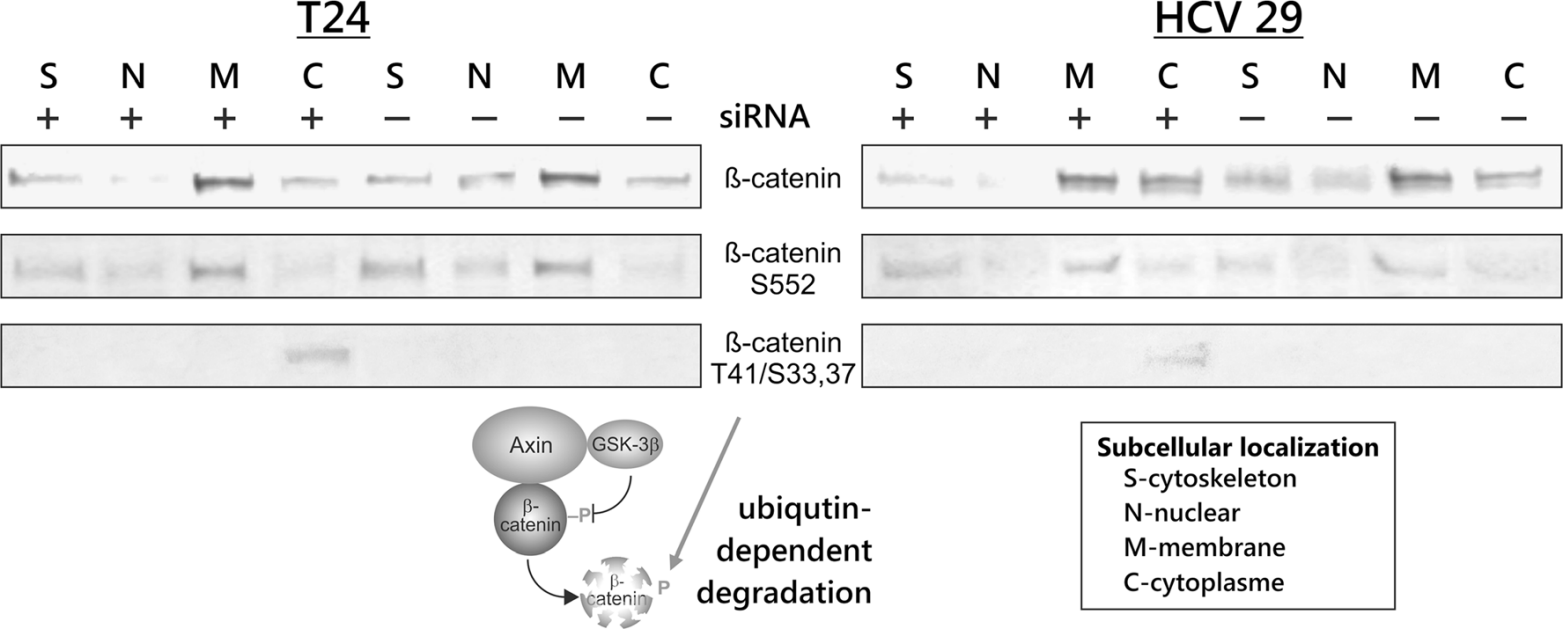
β-catenin expression in effect of ILK silencing. Various subcellular fractions of bladder cancer cells were analyzed for β-catenin expression by Western blotting. ILK silencing increases β-catenin degradation which is flagged for degradation by phosphorylation at S33, 37 T41. ILK regulates also the nuclear translocation and transcriptional activity of β-catenin. Cytoplasmic (*C*), nuclear (*N*), membrane (*M*), and cytoskeleton (*S*) proteins were extracted as described in “Materials and methods” section. Data were obtained from triplicate experiments

Because the key protein regulated by GSK-3β is β- catenin, we next examined the relationship between the lower phosphorylation of Ser 9 GSK-3β and the levels of total β-catenin and its nuclear accumulation. β-catenin is phosphorylated in its N-terminal domain by GSK-3β, which leads to its degradation by ubiquitination [5]. Knocking down ILK reduced the total level of β-catenin in both studied cell lines (Fig. 1). We detected increased GSK-3-mediated phosphorylation of β-catenin on S33, S37, and T41 after silencing of ILK, that is associated with the β-catenin destruction (Fig. 2). We also observed the reduction of nuclear accumulation of β-catenin in both cell lines after ILK depletion (Fig. 2).

Nuclear β-catenin combines with transcription factors of the TCF/LEF-1 family and promotes tumor progression, but nuclear translocation is not adequate for transcriptional activity of β-catenin. Phosphorylation of β-catenin at C-terminal residues Ser 552 mediated by Akt is necessary for its transcriptional activity [13]. This phosphorylation of β-catenin was reduced in the T24 transitional cancer cells and completely abolished in the HCV29 nonmalignant transitional epithelial cells of the urether upon ILK siRNA transfection (Fig. 1). The nuclear accumulation of β-catenin phosphorylated on S552 was suppressed (Fig. 2).

### ILK regulates cadherin switch

**Fig. 3:**
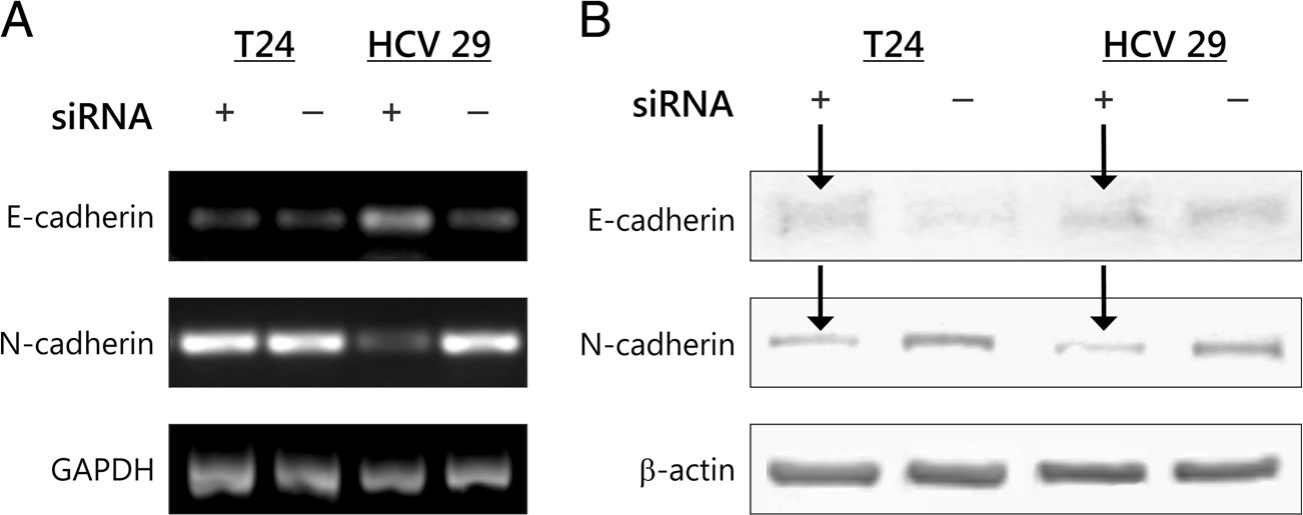
ILK regulates cadherin switch in bladder cells. **a**. E-cadherin and N-cadherin expressions on mRNA level were determined by reverse transcription-PCR 48 h after transfection. **b**. Inhibition of ILK expression by siRNA is sufficient also to cadherin switch on protein level, Western blotting. Total protein loading was determined by probing the membranes for β-actin. Shown are representative membranes of at least three independent experiments with similar results

**Fig. 4:**
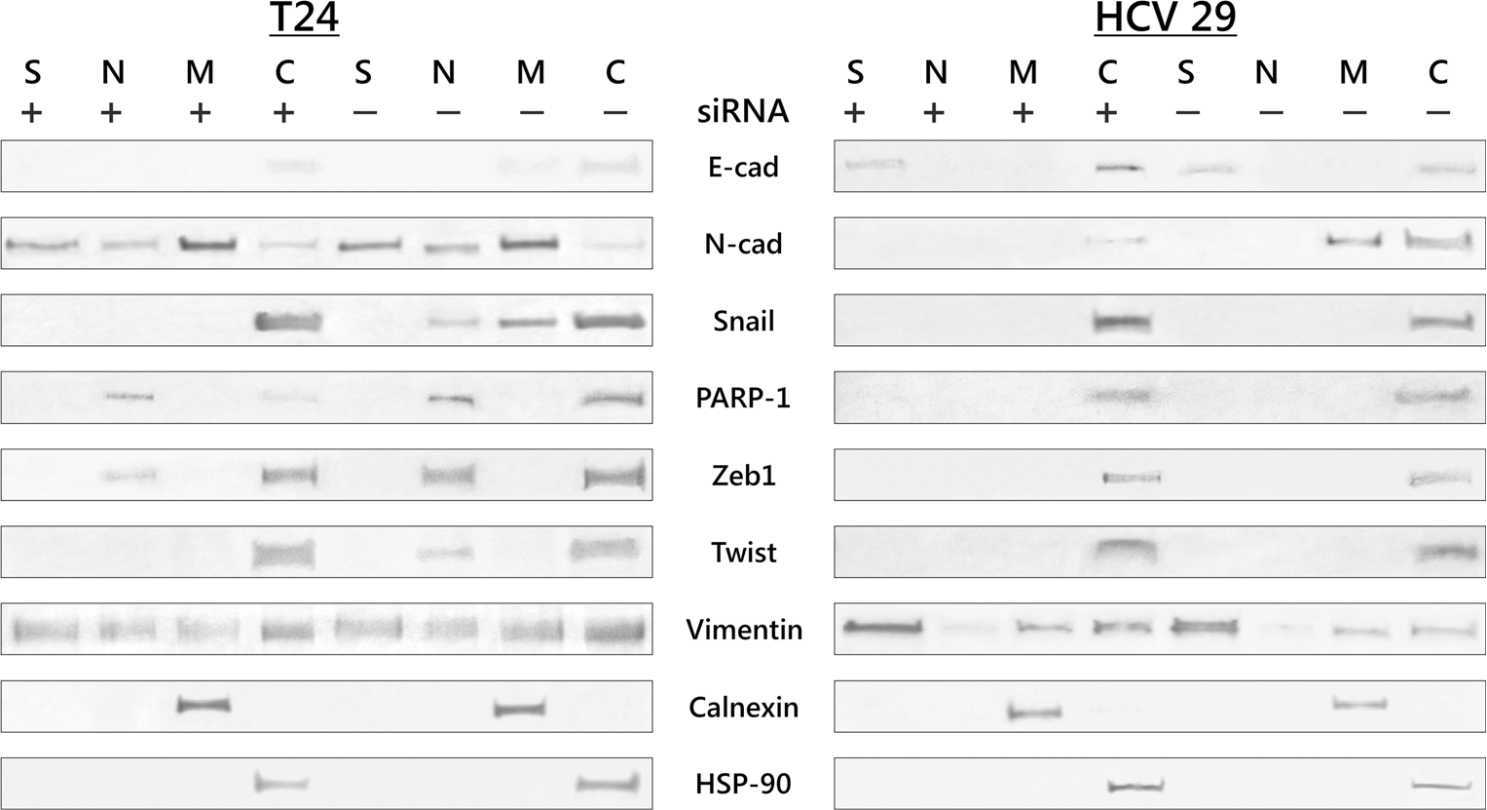
ILK regulates expression and cellular localization of key transcriptional factors and markers of EMT. Cytoplasmic (*C*), nuclear (*N*), membrane (*M*), and cytoskeleton (*S*) proteins were extracted as described in “Materials and methods” section. Calnexin and HSP-90 were used as marker proteins specific for the appropriate fraction. Data were obtained from triplicate experiments

Studied bladder cell line T24 expresses N-cadherin but no or little E-cadherin at the protein level, and a low amount on messenger RNA (mRNA) level (Fig. 3). HCV 29 cells express a lower level of both E- and N-cadherins. The increase of N-cadherin expression in bladder cancer has been shown to be important in cancer progression [14]. We checked whether the ILK signaling pathway was necessary for cadherin switch in bladder cell lines. As shown in Figs. 3 and 4, inhibition of ILK activity caused by siRNA was sufficient to downregulate the expression of N-cadherin on protein level in both cell lines and significant decrease on mRNA level in HCV29. In this study, the effects of re-expression of E-cadherin on mRNA and protein level were markedly higher in HCV29 compared with control nonsilencing RNA, and the re-expression of E-cadherin on protein level was slightly visible upon silencing of ILK in T24 bladder cancer cells (Figs. 3 and 4).

### siRNA-mediated knockdown of ILK inhibits nuclear translocation of key transcription factors including Snail, Twist, and Zeb

Genes of a family of transcriptional factors including Snail, Twist, and Zeb are called “EMT master genes”. The function of these transcription factors is regulated at the transcriptional, translational, and posttranslational levels [15]. Because nuclear accumulation of key transcriptional factors is one common EMT markers, we examined whether ILK signaling pathways result in production and intracellular localization of these factors. Western blot analysis of subcellular fraction (Fig. 4) showed that Snail, Twist-1, and Zeb-1 protein nuclear translocation was inhibited in T24 bladder cancer cells depleted of ILK. HCV29 nonmalignant transitional epithelial cells did not express detectable levels of any of the three transcriptional factors in nuclear fraction.

### ILK regulates PARP-1 expression

The nuclear protein PARP-1(poly-ADP-ribose polymerase 1), known to function as a DNA damage sensor and to play a role in DNA repair pathways, has recently been implicated in transcriptional regulation [16]. PARP-1 regulates transcription by modifying chromatin structure and through interaction with other transcription factors. The siRNA reduction of ILK expression had significant effects on PARP-1 protein levels in the bladder cancer cell line T24 (Fig. 4).

## Discussion

The epithelial-mesenchymal transition gives mesenchymal properties on epithelial cells and is closely linked with development of aggressive traits by cancer cells. A pivotal alteration that occurs during EMT is the “cadherin switch”, in which the expression of E-cadherin is substituted by abnormal expression of N-cadherin. This downregulation of epithelial cadherin is linked with the release of β-catenin, which then travels to the nucleus and activates transcription of other genes responsive for EMT.

A large amount of research demonstrate interactions between cadherins and integrins, suggesting a connection between signaling pathways both integrin and cadherin. Alexander et al. [17] presented that the induction of N-cadherin mRNA is dependent on β_1_ integrin in PC-3 prostate adenocarcinoma cell line. In addition, in mouse mammary epithelial cells, Shintani et al. [18] documented that upregulation of N-cadherin and EMT is initiated by collagen I receptors. Koenig et al. [19] evidenced that collagen type I by interaction with β_1_ integrins caused loss of E-cadherin-mediated cell-cell contacts and promoted proliferation of pancreatic carcinoma cells. Additionally, Kim et al. [20] reported that deletion of α_3_β_1_ integrin in epithelial cells prevented EMT response to TGF-β. Our data demonstrate a new molecular mechanism in which ILK can regulate the “cadherin switch”. In our earlier work, we suggested that ILK may mediate cross-talk between adhesion molecules in melanoma cells and the control of cadherin expression by integrins might be a general feature common to all motile cells [3].

It is known that ILK suppresses E-cadherin expression by controling the expression of its suppressor, Snail [5]. Overexpression of ILK was associated with the expression of the E-cadherin repressor Snail and N-cadherin in pancreatic adenocarcinoma [21]. Matsui et al. [11] presented that ILK overexpression correlates with bladder invasiveness via the control of E-cadherin and matrix metalloprotease 9 (MMP-9). We also noticed that ILK can regulate expression of MMP-9 (Supplementary Fig. 1). Activation of ILK causes the regulation of numerous signaling pathways that in turn regulate EMT. We showed that knockdown of ILK diminished phosphorylation of downstream signaling target protein kinase Akt and glycogen synthase kinase -3β (GSK-3β), whereas the levels of Akt and GSK-3β protein remained almost unchanged. GSK activity is regulated through both serine and tyrosine phosphorylation [22]. Akt phosphorylates GSK-3β at Ser 9, leading to inactivation of its kinase activity, and ILK can phosphorylate GSK-3β at Ser 9 in Akt-independent way [23]. Luo et al. [24] demonstrated that ILK knockdown inhibits EMT through inhibition of GSK-3β phosphorylation at Ser 9. On the other hand, actions which promote cell death, such as growth factor removal, stimulate kinase activity by increasign phosphorylation within the catalytic domain at Tyr 216. On top of it, Meares and Jope [12] presented that phosphorylation of Tyr 216 of GSK-3β is responsible for the nuclear distribution of GSK-3β. They demonstrated that upon mutation of Tyr 216 to Phe, less GSK-3β is shifted to the nucleus. ILK knockdown in this study decreased the levels of phospho-Ser 9 GSK-3β form with a significant increase in the level of GSK-3β phospho-Tyr 216 form. We had similar results after silencing the ILK in melanoma cells [3]. This was accompanied by significant decrease in expression of β-catenin, compared to the control. It is well known that Akt, GSK-3, and β-catenin are important mediators of ILK signaling. Yang et al. [25] described the similar observation that ILK can regulate β-catenin accumulation in the nucleus and its activation in epithelial cells or intercellular communication via gap junctions. β-catenin plays also a critical structural role in cadherin-based adhesion. Although E-cadherin-bound β-catenin is relatively stable, the availability of β-catenin for binding cadherins is regulated by its phosphorylation [13]. The cytosolic pool of β-catenin is also regulated by a phosphorylation-based mechanism. β-catenin is phosphorylated by GSK-3β at Ser 33, Ser 37, and Thr 47 within the N-terminal domain, which leads to its degradation by the ubiquitin/proteasome pathway. Our data show that siRNA-mediated depletion of ILK caused a phosphorylation on N-terminal residues and decrease in the total level of β-catenin in both bladder cell lines. These studies indicate ILK as a critical factor for nuclear translocation of β-catenin and activation of transcription factors, which upregulate the expression of oncogenic and mesenchymal genes in bladder cells. Furthermore, Fang et al. [13] documented that β-catenin accumulation in the nucleus is not sufficient for β-catenin/TCF transcriptional activity. They showed that cells stably transfected with β-catenin S552A had a lower transcriptional activity that has been linked to tumor cell invasiveness. They proved that phosphorylation of β-catenin at C-terminal residues Ser 552 by Akt was necessary for the promotion of β-catenin transcriptional activity. Additionally, Miyabayashi and collegues [26] suggested that phosphorylation of β-catenin may dictate to which particular co-activators bound to β-catenin and as a result, target genes can be activated. Our results also indicate the essential role of phosphorylation in the regulation of β-catenin function, based on the data that phosphorylation of β-catenin at Ser 552 is eliminated upon ILK silencing in both cell lines. β-catenin transcriptional activity has been associated with tumor progression, and nuclear translocation of β-catenin is very often used as an EMT marker. Our observation that ILK regulates expression of either EMT-inducing transcription factors Twsit-1, Zeb-1, and Snail indicates that ILK may function as upstream regulator of EMT associated signaling networks. Twist-1, Zeb-1, and Snail are zinc-finger transcriptional repressors that bind directly to the E-boxes of the promoter of the E-cadherin coding gene, and Twist-1 has been suggested as the major regulator of N-cadherin expression during gastrulation in *Drosophila* and promoting the N-cadherin expression in gastric cancers [27]. Our data indicated that regulation of cadherin switch by ILK pathway was mediated by the transcriptional repressors, as the loss of nuclear translocation of Snail, Zeb-1, and Twist-1 leads to upregulation of E-cadherin. E-cadherin re-expression also supports relocalization of β-catenin from the nucleus to the plasma membrane.

Several investigators reported new insights to control the production, stability, and intracellular localization of these transcriptional repressors [9, 15, 28, 29].

McPhee et al. [9] took notice that PARP-1 binds the SIRE sequence in Snail-1 promoter only in the presence but not the absence of ILK. They also showed that loss of ILK expression in prostate cancer cells had no effect on PARP-1 protein levels. The date presented in this study indicated that the amount of PARP-1 protein was reduced when ILK expression was inhibited. Snail activity can also be controlled by GSK-3. Zhou et al. [28] reported that Snail-1 is phosphorylated by GSK-3 on two distinct motifs. Phosphorylation of two serines in the first motif leads to Snail ubiquitination, whereas phosphorylation of four serines on the second motif directs nuclear export. Mutation of all six serines increased the half-life of the Snail which resides exclusively in the nucleus to induce EMT. We did not observe detectable levels of Snail in nuclear fraction of HCV29 nonmalignant transitional epithelial cells, and we have shown inhibition of nuclear translocation of Snail in T24 bladder transitional cancer cells after silencing of ILK. Thus, Snail and GSK-3β together function as a molecular switch for many ILK signaling pathways that lead to EMT. We demonstrated that targeting ILK could control also the nuclear translocation of Zeb-1 and Twist in T24 bladder transitional cancer cells. Wu et al. [29] suggested that Zeb-1 expression is necessary for transitional cancer cell invasion and distant metastasis in bladder cancer and that β-catenin induces Zeb-1 transcription. They also found that Zeb-1 could regulate expression of cytokeratins, vimentin, and MMP-2 but not N-cadherin expression. Although the mechanisms regulating the aberrant expression of N-cadherin in carcinoma progression remain unknown, the Twist-1 expression has been indicated to be necessary for N-cadherin expression during gastrulation in *Drosophila.* Yang and colleagues suggested that Twist expression was sufficient to induce in vitro EMT [27], and Alexander and colleagues demonstrated that integrin-mediated adhesion is involved in the Twist-1 nuclear translocation and is necessary for N-cadherin expression in PC-3 prostate carcinoma cells [17]. Recently, Yang and coauthors discovered that knocking down ILK or inhibiting FAK, MAPK/ERK, or PI3K/Akt signaling suppressed Twist-induced EMT [30]. Notably, they found that integrin β_1_ acts as a core regulator in this network. Our date confirmed that ILK is implicated in nuclear translocation of Twist, because Twist was absent in the nucleus after ILK silencing in comparison to control cells in T24 cells. Although the total level of Twist-1 did not change significantly, Twist protein was present only in cytoplasm fraction. Our results showed that in T24 cells, the alteration of protein level was more significant than that of the mRNA of N-cadherin, suggesting posttranscriptional regulation of N-cadherin by Twist, as it has been recently observed in gastric cancer [27]. Twist protein is able to form homo and heterodimers, and posttranslational modification, such as phosphorylation, can alter its interaction with other proteins and the binding to DNA. In our previous work, after ILK silencing in melanoma cells, we observed a decrease in expression of N-cadherin but only at the protein level; the re-expression of E-cadherin on mRNA or protein level was not observed [3]. The specific mechanism through ILK modulates N-cadherin expression is not clear. Presented results show that ILK pathway regulates the cadherin switch of bladder cancer through multiple mechanisms, including transcriptional and posttranslational regulation.

It is difficult to indicate a common pathway controlling EMT under ILK regulation, assuming that various pathways may be involved and may vary in different cell types.

## Electronic supplementary material

Supplementary Figure 1 Knockdown of ILK leads to decreased expression of matrix metalloprotease 9 (MMP-9). Bladder cells were transfected with nonsilencing control siRNA or three different 21 bp double-stranded siRNA targeting the ILK. Forty-eight hours after transfection, protein expression was analyzed by Western blot. Antibody from cell signaling technology (#3852), which detects full length (proenzyme, 92 kDa) and cleaved (active enzyme, 84 kDa) MMP-9 was used. *Arrow* indicates the active MMP-9 form. Total protein loading was determined by probing the membranes for β-actin. Densitometry was used to normalize to β-actin protein level and for quantitative comparison after siRNA knockdown. Presented are representative membranes of at least three independent experiments with similar results (GIF 138 kb)

High resolution image (TIFF 242 kb)
