# Supplementary figures and images for: Integrin-linked kinase regulates cadherin switch in bladder cancer
Source: Tumour Biol. 2016 Sep 28;37(11):15185–91. doi: 10.1007/s13277-016-5354-x (PMC5126188; doi:10.1007/s13277-016-5354-x)

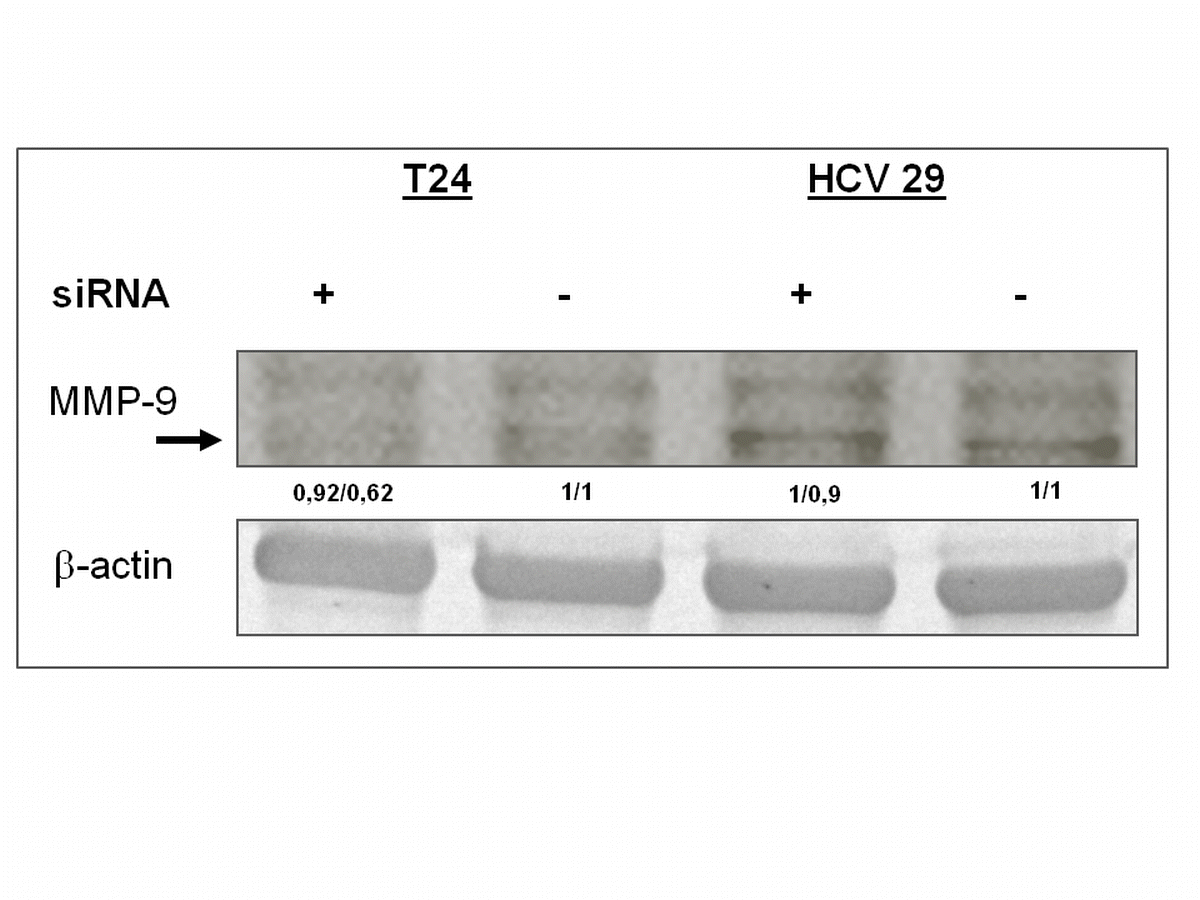

Supplement: Supplementary file 1 — Knockdown of ILK leads to decreased expression of matrix metalloprotease 9 (MMP-9). Bladder cells were transfected with nonsilencing control siRNA or three different 21 bp double-stranded siRNA targeting the ILK. Forty-eight hours after transfection, protein expression was analyzed by Western blot. Antibody from cell signaling technology (#3852), which detects full length (proenzyme, 92 kDa) and cleaved (active enzyme, 84 kDa) MMP-9 was used. Arrow indicates the active MMP-9 form. Total protein loading was determined by probing the membranes for β-actin. Densitometry was used to normalize to β-actin protein level and for quantitative comparison after siRNA knockdown. Presented are representative membranes of at least three independent experiments with similar results (GIF 138 kb) [file 13277_2016_5354_Fig5_ESM.gif]

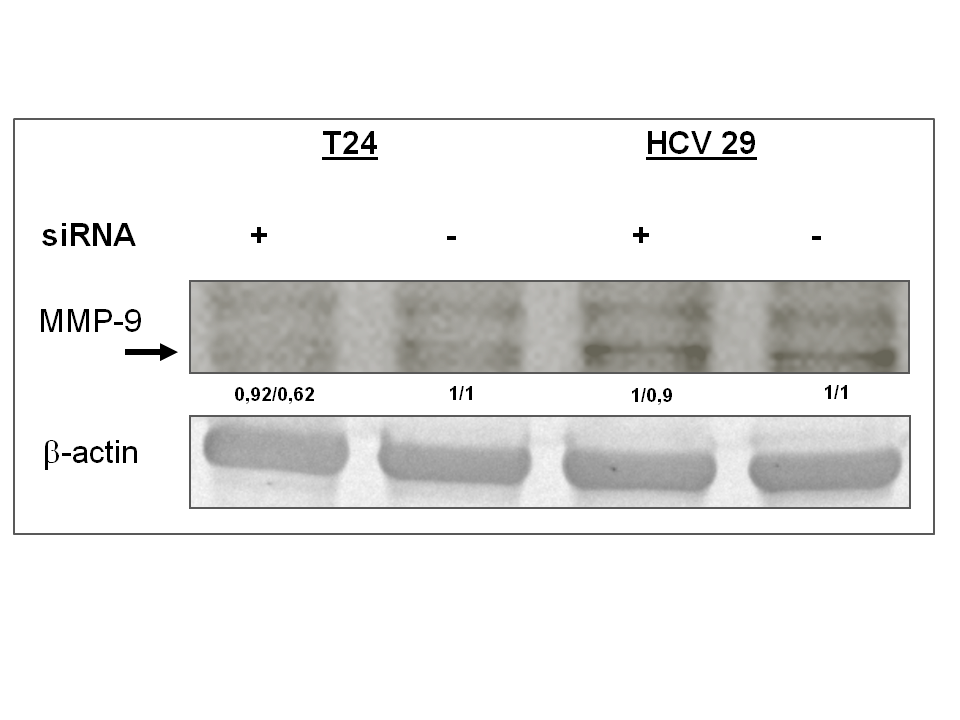

Supplement: Supplementary file 2 — High resolution image (TIFF 242 kb) [file 13277_2016_5354_MOESM1_ESM.tif]
